# Deciphering the influence: academic stress and its role in shaping learning approaches among nursing students: a cross-sectional study

**DOI:** 10.1186/s12912-024-01885-1

**Published:** 2024-04-17

**Authors:** Rawhia Salah Dogham, Heba Fakieh Mansy Ali, Asmaa Saber Ghaly, Nermine M. Elcokany, Mohamed Mahmoud Seweid, Ayman Mohamed El-Ashry

**Affiliations:** 1 Nursing Education, Faculty of Nursing, Alexandria University, Alexandria, Egypt; 2 Critical Care & Emergency Nursing, Faculty of Nursing, Alexandria University, Alexandria, Egypt; 3 Obstetrics and Gynecology Nursing, Faculty of Nursing, Alexandria University, Alexandria, Egypt; 4 Faculty of Nursing, Beni-Suef University, Beni-Suef, Egypt; 5 Psychiatric and Mental Health Nursing, Faculty of Nursing, Alexandria University, Alexandria, Egypt

**Keywords:** Academic stress, Education, Learning approaches, Nursing students

## Abstract

**Background:**

Nursing education presents unique challenges, including high levels of academic stress and varied learning approaches among students. Understanding the relationship between academic stress and learning approaches is crucial for enhancing nursing education effectiveness and student well-being.

**Aim:**

This study aimed to investigate the prevalence of academic stress and its correlation with learning approaches among nursing students.

**Design and Method:**

A cross-sectional descriptive correlation research design was employed. A convenient sample of 1010 nursing students participated, completing socio-demographic data, the Perceived Stress Scale (PSS), and the Revised Study Process Questionnaire (R-SPQ-2 F).

**Results:**

Most nursing students experienced moderate academic stress (56.3%) and exhibited moderate levels of deep learning approaches (55.0%). Stress from a lack of professional knowledge and skills negatively correlates with deep learning approaches (r = -0.392) and positively correlates with surface learning approaches (r = 0.365). Female students showed higher deep learning approach scores, while male students exhibited higher surface learning approach scores. Age, gender, educational level, and academic stress significantly influenced learning approaches.

**Conclusion:**

Academic stress significantly impacts learning approaches among nursing students. Strategies addressing stressors and promoting healthy learning approaches are essential for enhancing nursing education and student well-being.

**Nursing implication:**

Understanding academic stress’s impact on nursing students’ learning approaches enables tailored interventions. Recognizing stressors informs strategies for promoting adaptive coping, fostering deep learning, and creating supportive environments. Integrating stress management, mentorship, and counseling enhances student well-being and nursing education quality.

## Introduction

Nursing education is a demanding field that requires students to acquire extensive knowledge and skills to provide competent and compassionate care. Nursing education curriculum involves high-stress environments that can significantly impact students’ learning approaches and academic performance [1, 2]. Numerous studies have investigated learning approaches in nursing education, highlighting the importance of identifying individual students’ preferred approaches. The most studied learning approaches include deep, surface, and strategic approaches. Deep learning approaches involve students actively seeking meaning, making connections, and critically analyzing information. Surface learning approaches focus on memorization and reproducing information without a more profound understanding. Strategic learning approaches aim to achieve high grades by adopting specific strategies, such as memorization techniques or time management skills [3–5].

Nursing education stands out due to its focus on practical training, where the blend of academic and clinical coursework becomes a significant stressor for students, despite academic stress being shared among all university students [6–8]. Consequently, nursing students are recognized as prone to high-stress levels. Stress is the physiological and psychological response that occurs when a biological control system identifies a deviation between the desired (target) state and the actual state of a fitness-critical variable, whether that discrepancy arises internally or externally to the human [9]. Stress levels can vary from objective threats to subjective appraisals, making it a highly personalized response to circumstances. Failure to manage these demands leads to stress imbalance [10].

Nursing students face three primary stressors during their education: academic, clinical, and personal/social stress. Academic stress is caused by the fear of failure in exams, assessments, and training, as well as workload concerns [11]. Clinical stress, on the other hand, arises from work-related difficulties such as coping with death, fear of failure, and interpersonal dynamics within the organization. Personal and social stressors are caused by an imbalance between home and school, financial hardships, and other factors. Throughout their education, nursing students have to deal with heavy workloads, time constraints, clinical placements, and high academic expectations. Multiple studies have shown that nursing students experience higher stress levels compared to students in other fields [12–14].

Research has examined the relationship between academic stress and coping strategies among nursing students, but no studies focus specifically on the learning approach and academic stress. However, existing literature suggests that students interested in nursing tend to experience lower levels of academic stress [7]. Therefore, interest in nursing can lead to deep learning approaches, which promote a comprehensive understanding of the subject matter, allowing students to feel more confident and less overwhelmed by coursework and exams. Conversely, students employing surface learning approaches may experience higher stress levels due to the reliance on memorization [3].

Understanding the interplay between academic stress and learning approaches among nursing students is essential for designing effective educational interventions. Nursing educators can foster deep learning approaches by incorporating active learning strategies, critical thinking exercises, and reflection activities into the curriculum [15]. Creating supportive learning environments encouraging collaboration, self-care, and stress management techniques can help alleviate academic stress. Additionally, providing mentorship and counselling services tailored to nursing students’ unique challenges can contribute to their overall well-being and academic success [16–18].

Despite the scarcity of research focusing on the link between academic stress and learning methods in nursing students, it’s crucial to identify the unique stressors they encounter. The intensity of these stressors can be connected to the learning strategies employed by these students. Academic stress and learning approach are intertwined aspects of the student experience. While academic stress can influence learning approaches, the choice of learning approach can also impact the level of academic stress experienced. By understanding this relationship and implementing strategies to promote healthy learning approaches and manage academic stress, educators and institutions can foster an environment conducive to deep learning and student well-being.

Hence, this study aims to investigate the correlation between academic stress and learning approaches experienced by nursing students.

## Study objectives

- Assess the levels of academic stress among nursing students.
- Assess the learning approaches among nursing students.
- Identify the relationship between academic stress and learning approach among nursing students.
- Identify the effect of academic stress and related factors on learning approach and among nursing students.

## Materials and methods

### Research design

A cross-sectional descriptive correlation research design adhering to the STROBE guidelines was used for this study.

### Setting

A research project was conducted at Alexandria Nursing College, situated in Egypt. The college adheres to the national standards for nursing education and functions under the jurisdiction of the Egyptian Ministry of Higher Education. Alexandria Nursing College comprises nine specialized nursing departments that offer various nursing specializations. These departments include Nursing Administration, Community Health Nursing, Gerontological Nursing, Medical-Surgical Nursing, Critical Care Nursing, Pediatric Nursing, Obstetric and Gynecological Nursing, Nursing Education, and Psychiatric Nursing and Mental Health. The credit hour system is the fundamental basis of both undergraduate and graduate programs. This framework guarantees a thorough evaluation of academic outcomes by providing an organized structure for tracking academic progress and conducting analyses.

### Participants and sample size calculation

The researchers used the Epi Info 7 program to calculate the sample size. The calculations were based on specific parameters such as a population size of 9886 students for the academic year 2022–2023, an expected frequency of 50%, a maximum margin of error of 5%, and a confidence coefficient of 99.9%. Based on these parameters, the program indicated that a minimum sample size of 976 students was required. As a result, the researchers recruited a convenient sample of 1010 nursing students from different academic levels during the 2022–2023 academic year [19]. This sample size was larger than the minimum required, which could help to increase the accuracy and reliability of the study results. Participation in the study required enrollment in a nursing program and voluntary agreement to take part. The exclusion criteria included individuals with mental illnesses based on their response and those who failed to complete the questionnaires.

### Tools

#### Tool one

socio-demographic data that include students’ age, sex, educational level, hours of sleep at night, hours spent studying, and GPA from the previous semester.

#### Tool two: the perceived stress scale (PSS)

It was initially created by Sheu et al. (1997) to gauge the level and nature of stress perceived by nursing students attending Taiwanese universities [20]. It comprises 29 items rated on a 5-point Likert scale, where (0 = never, 1 = rarely, 2 = sometimes, 3 = reasonably often, and 4 = very often), with a total score ranging from 0 to 116. The cut-off points of levels of perceived stress scale according to score percentage were low < 33.33%, moderate 33.33–66.66%, and high more than 66.66%. Higher scores indicate higher stress levels. The items are categorized into six subscales reflecting different sources of stress. The first subscale assesses “stress stemming from lack of professional knowledge and skills” and includes 3 items. The second subscale evaluates “stress from caring for patients” with 8 items. The third subscale measures “stress from assignments and workload” with 5 items. The fourth subscale focuses on “stress from interactions with teachers and nursing staff” with 6 items. The fifth subscale gauges “stress from the clinical environment” with 3 items. The sixth subscale addresses “stress from peers and daily life” with 4 items. El-Ashry et al. (2022) reported an excellent internal consistency reliability of 0.83 [21]. Two bilingual translators translated the English version of the scale into Arabic and then back-translated it into English by two other independent translators to verify its accuracy. The suitability of the translated version was confirmed through a confirmatory factor analysis (CFA), which yielded goodness-of-fit indices such as a comparative fit index (CFI) of 0.712, a Tucker-Lewis index (TLI) of 0.812, and a root mean square error of approximation (RMSEA) of 0.100.

#### Tool three: revised study process questionnaire (R-SPQ-2 F)

It was developed by Biggs et al. (2001). It examines deep and surface learning approaches using only 20 questions; each subscale contains 10 questions [22]. On a 5-point Likert scale ranging from 0 (never or only rarely true of me) to 4 (always or almost always accurate of me). The total score ranged from 0 to 80, with a higher score reflecting more deep or surface learning approaches. The cut-off points of levels of revised study process questionnaire according to score percentage were low < 33%, moderate 33–66%, and high more than 66%. Biggs et al. (2001) found that Cronbach alpha value was 0.73 for deep learning approach and 0.64 for the surface learning approach, which was considered acceptable. Two translators fluent in English and Arabic initially translated a scale from English to Arabic. To ensure the accuracy of the translation, they translated it back into English. The translated version’s appropriateness was evaluated using a confirmatory factor analysis (CFA). The CFA produced several goodness-of-fit indices, including a Comparative Fit Index (CFI) of 0.790, a Tucker-Lewis Index (TLI) of 0.912, and a Root Mean Square Error of Approximation (RMSEA) of 0.100. Comparative Fit Index (CFI) of 0.790, a Tucker-Lewis Index (TLI) of 0.912, and a Root Mean Square Error of Approximation (RMSEA) of 0.100.

### Procedures

#### Ethical considerations

The Alexandria University College of Nursing’s Research Ethics Committee provided ethical permission before the study’s implementation. Furthermore, pertinent authorities acquired ethical approval at participating nursing institutions. The vice deans of the participating institutions provided written informed consent attesting to institutional support and authority. By giving written informed consent, participants confirmed they were taking part voluntarily. Strict protocols were followed to protect participants’ privacy during the whole investigation. The obtained personal data was kept private and available only to the study team. Ensuring participants’ privacy and anonymity was of utmost importance.

#### Tools validity

The researchers created tool one after reviewing pertinent literature. Two bilingual translators independently translated the English version into Arabic to evaluate the applicability of the academic stress and learning approach tools for Arabic-speaking populations. To assure accuracy, two additional impartial translators back-translated the translation into English. They were also assessed by a five-person jury of professionals from the education and psychiatric nursing departments. The scales were found to have sufficiently evaluated the intended structures by the jury.

#### Pilot study

A preliminary investigation involved 100 nursing student applicants, distinct from the final sample, to gauge the efficacy, clarity, and potential obstacles in utilizing the research instruments. The pilot findings indicated that the instruments were accurate, comprehensible, and suitable for the target demographic. Additionally, Cronbach’s Alpha was utilized to further assess the instruments’ reliability, demonstrating internal solid consistency for both the learning approaches and academic stress tools, with values of 0.91 and 0.85, respectively.

#### Data collection

The researchers convened with each qualified student in a relaxed, unoccupied classroom in their respective college settings. Following a briefing on the study’s objectives, the students filled out the datasheet. The interviews typically lasted 15 to 20 min.

#### Data analysis

The data collected were analyzed using IBM SPSS software version 26.0. Following data entry, a thorough examination and verification were undertaken to ensure accuracy. The normality of quantitative data distributions was assessed using Kolmogorov-Smirnov tests. Cronbach’s Alpha was employed to evaluate the reliability and internal consistency of the study instruments. Descriptive statistics, including means (M), standard deviations (SD), and frequencies/percentages, were computed to summarize academic stress and learning approaches for categorical data. Student’s t-tests compared scores between two groups for normally distributed variables, while One-way ANOVA compared scores across more than two categories of a categorical variable. Pearson’s correlation coefficient determined the strength and direction of associations between customarily distributed quantitative variables. Hierarchical regression analysis identified the primary independent factors influencing learning approaches. Statistical significance was determined at the 5% (p < 0.05).

## Results

Table 1 presents socio-demographic data for a group of 1010 nursing students. The age distribution shows that 38.8% of the students were between 18 and 21 years old, 32.9% were between 21 and 24 years old, and 28.3% were between 24 and 28 years old, with an average age of approximately 22.79. Regarding gender, most of the students were female (77%), while 23% were male. The students were distributed across different educational years, a majority of 34.4% in the second year, followed by 29.4% in the fourth year. The students’ hours spent studying were found to be approximately two-thirds (67%) of the students who studied between 3 and 6 h. Similarly, sleep patterns differ among the students; more than three-quarters (77.3%) of students sleep between 5- to more than 7 h, and only 2.4% sleep less than 2 h per night. Finally, the student’s Grade Point Average (GPA) from the previous semester was also provided. 21% of the students had a GPA between 2 and 2.5, 40.9% had a GPA between 2.5 and 3, and 38.1% had a GPA between 3 and 3.5.

**Fig. 1:**
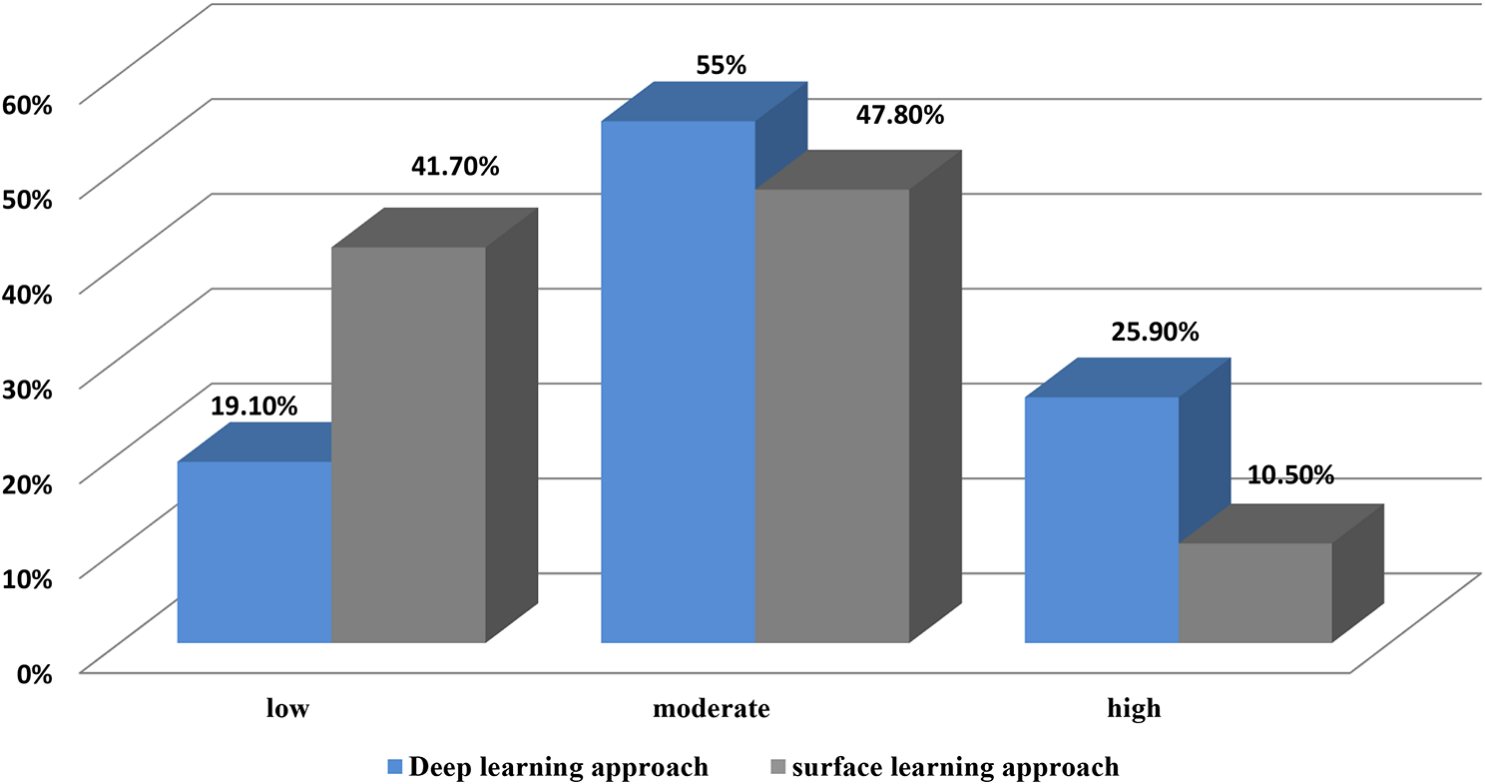
Nursing students? levels of learning approach (N=1010)

Figure 1 provides the learning approach level among nursing students. In terms of learning approach, most students (55.0%) exhibited a moderate level of deep learning approach, followed by 25.9% with a high level and 19.1% with a low level. The surface learning approach was more prevalent, with 47.8% of students showing a moderate level, 41.7% showing a low level, and only 10.5% exhibiting a high level.

**Table 1:** Distribution of nursing students according to their socio- demographic data (N = 1010)

Socio-demographic data	Number	Percentage (%)
**Age**		
18 ≤ 21	392	38.8
21 ≤ 24	332	32.9
24 ≤ 28	286	28.3
Mean ± SD.	22.79 ± 4.69	
**Gender**		
Male	232	23.0
Female	778	77.0
**Educational**		
1st	149	14.8
2nd	347	34.4
3rd	217	21.5
4th	297	29.4
**Hours spent for studying**		
Less than 2 h	109	10.8
3–4 h	355	35.1
5–6 h	323	32.0
More than 7 h	223	22.1
**Hours of sleep / night**		
Less than 2 h	24	2.4
3–4 h	174	17.2
5–6 h	556	55.0
More than 7 h	256	25.3
**GPA of previous semester**		
2–2.5	212	21.0
2.5–3	413	40.9
3–3.5	385	38.1

**Fig. 2:**
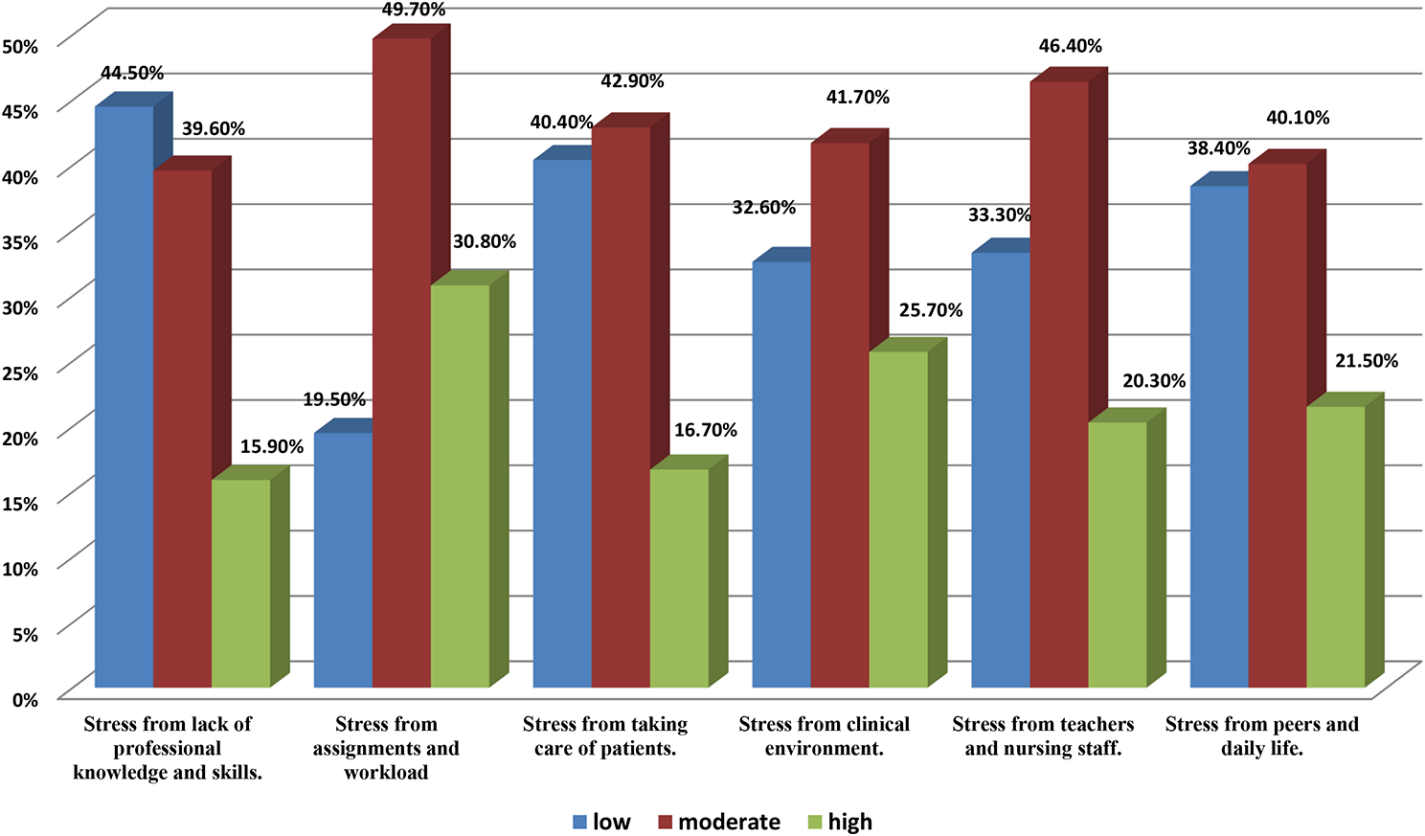
Nursing students? levels of academic stress subtypes (N=1010)

Figure 2 provides the types of academic stress levels among nursing students. Among nursing students, various stressors significantly impact their academic experiences. Foremost among these stressors are the pressure and demands associated with academic assignments and workload, with 30.8% of students attributing their high stress levels to these factors. Challenges within the clinical environment are closely behind, contributing significantly to high stress levels among 25.7% of nursing students. Interactions with peers and daily life stressors also weigh heavily on students, ranking third among sources of high stress, with 21.5% of students citing this as a significant factor. Similarly, interaction with teachers and nursing staff closely follow, contributing to high-stress levels for 20.3% of nursing students. While still significant, stress from taking care of patients ranks slightly lower, with 16.7% of students reporting it as a significant factor contributing to their academic stress. At the lowest end of the ranking, but still notable, is stress from a perceived lack of professional knowledge and skills, with 15.9% of students experiencing high stress in this area.

**Fig. 3:**
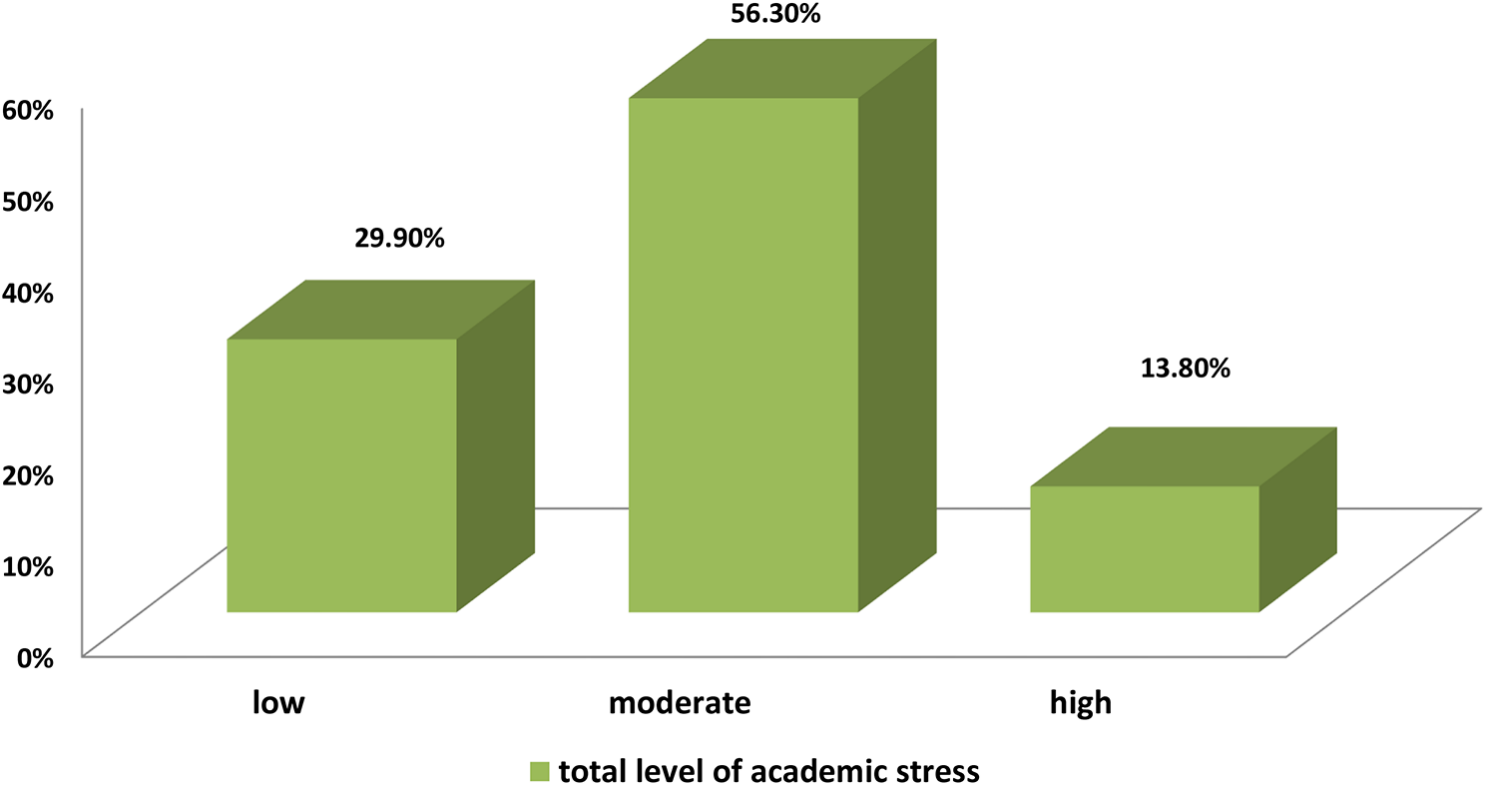
Nursing students? levels of total academic stress (N=1010)

Figure 3 provides the total levels of academic stress among nursing students. The majority of students experienced moderate academic stress (56.3%), followed by those experiencing low academic stress (29.9%), and a minority experienced high academic stress (13.8%).

Table 2 displays the correlation between academic stress subscales and deep and surface learning approaches among 1010 nursing students. All stress subscales exhibited a negative correlation regarding the deep learning approach, indicating that the inclination toward deep learning decreases with increasing stress levels. The most significant negative correlation was observed with stress stemming from the lack of professional knowledge and skills (r=-0.392, p < 0.001), followed by stress from the clinical environment (r=-0.109, p = 0.001), stress from assignments and workload (r=-0.103, p = 0.001), stress from peers and daily life (r=-0.095, p = 0.002), and stress from patient care responsibilities (r=-0.093, p = 0.003). The weakest negative correlation was found with stress from interactions with teachers and nursing staff (r=-0.083, p = 0.009). Conversely, concerning the surface learning approach, all stress subscales displayed a positive correlation, indicating that heightened stress levels corresponded with an increased tendency toward superficial learning. The most substantial positive correlation was observed with stress related to the lack of professional knowledge and skills (r = 0.365, p < 0.001), followed by stress from patient care responsibilities (r = 0.334, p < 0.001), overall stress (r = 0.355, p < 0.001), stress from interactions with teachers and nursing staff (r = 0.262, p < 0.001), stress from assignments and workload (r = 0.262, p < 0.001), and stress from the clinical environment (r = 0.254, p < 0.001). The weakest positive correlation was noted with stress stemming from peers and daily life (r = 0.186, p < 0.001).

**Table 2:** Correlation between academic stress subscales and deep and superficial learning approach among nursing students (N = 1010)

Academic stress subscales		Learning approach	
		Deep approach	Superficial approach
Stress from lack of professional knowledge and skills	**r**	-0.392*	0.365*
	**p**	< 0.001*	< 0.001*
Stress from assignments and workload	**r**	-0.103*	0.262*
	**p**	0.001*	< 0.001*
Stress from taking care of patients	**r**	-0.093*	0.334*
	**p**	0.003*	< 0.001*
Stress from clinical environment	**r**	-0.109*	0.254*
	**p**	0.001*	< 0.001*
Stress from teachers and nursing staff	**r**	-0.083*	0.262*
	**p**	0.009*	< 0.001*
Stress from peers and daily life	**r**	-0.095*	0.186*
	**p**	0.002*	< 0.001*
Overall	**r**	-0.159*	0.355*
	**p**	< 0.001*	< 0.001*

r: Pearson correlation coefficient

*: Statistically significant at p ≤ 0.05

Table 3 outlines the association between the socio-demographic characteristics of nursing students and their deep and surface learning approaches. Concerning age, statistically significant differences were observed in deep and surface learning approaches (F = 3.661, p = 0.003 and F = 7.983, p < 0.001, respectively). Gender also demonstrated significant differences in deep and surface learning approaches (t = 3.290, p = 0.001 and t = 8.638, p < 0.001, respectively). Female students exhibited higher scores in the deep learning approach (31.59 ± 8.28) compared to male students (29.59 ± 7.73), while male students had higher scores in the surface learning approach (29.97 ± 7.36) compared to female students (24.90 ± 7.97). Educational level exhibited statistically significant differences in deep and surface learning approaches (F = 5.599, p = 0.001 and F = 17.284, p < 0.001, respectively). Both deep and surface learning approach scores increased with higher educational levels. The duration of study hours demonstrated significant differences only in the surface learning approach (F = 3.550, p = 0.014), with scores increasing as study hours increased. However, no significant difference was observed in the deep learning approach (F = 0.861, p = 0.461). Hours of sleep per night and GPA from the previous semester did not exhibit statistically significant differences in deep or surface learning approaches.

**Table 3:** Relation between nursing students’ socio- demographic data and the study variables (N = 1010)

Socio-demographic data	Deep Learning approach	Surface Learning approach
**Age**		
18 ≤ 21	29.65 ± 7.11	24.43 ± 6.88
21 ≤ 24	31.00 ± 8.16	27.17 ± 8.17
24 ≤ 28	34.76 ± 10.46	26.02 ± 9.54
**F (p)**	3.661* (0.003*)	7.983* (< 0.001*)
**Gender**		
Male	29.59 ± 7.73	29.97 ± 7.36
Female	31.59 ± 8.28	24.90 ± 7.97
**t(p)**	3.290* (0.001*)	8.638* (< 0.001*)
**Educational level**		
1–2	31.21 ± 7.35	22.23 ± 5.83
3–4	29.85 ± 8.15	25.67 ± 7.43
5–6	31.29 ± 7.68	27.13 ± 8.01
7–8	32.47 ± 8.80	27.68 ± 9.23
**F (p)**	5.599* (0.001*)	17.284* (< 0.001*)
**Hours spent for studying**		
Less than 2 h	30.64 ± 8.10	28.42 ± 7.06
3–4 h	30.72 ± 8.55	25.82 ± 8.16
5–6 h	31.36 ± 7.86	25.61 ± 7.72
More than 7 h	31.70 ± 8.14	25.97 ± 8.91
**F (p)**	**0.861 (0.461)**	3.550* (0.014*)
**Hours of sleep / night**		
Less than 2 h	28.38 ± 10.97	26.50 ± 8.79
3–4 h	30.85 ± 8.62	25.92 ± 7.82
5–6 h	31.40 ± 8.02	25.78 ± 8.14
More than 7 h	30.99 ± 7.96	26.75 ± 8.20
**F (p)**	**1.202 (0.308)**	**0.892 (0.445)**
**GPA of previous semester**		
2–2.5	30.95 ± 7.97	26.26 ± 7.80
2.5–3	31.33 ± 8.11	25.91 ± 8.34
3–3.5	31.02 ± 8.42	26.12 ± 8.06
**F (p)**	**0.213 (0.808)**	**0.145 (0.865)**

F: One way ANOVA test

*: Statistically significant at p ≤ 0.05

Table 4 presents a multivariate linear regression analysis examining the factors influencing the learning approach among 1110 nursing students. The deep learning approach was positively influenced by age, gender (being female), educational year level, and stress from teachers and nursing staff, as indicated by their positive coefficients and significant p-values (p < 0.05). However, it was negatively influenced by stress from a lack of professional knowledge and skills. The other factors do not significantly influence the deep learning approach. On the other hand, the surface learning approach was positively influenced by gender (being female), educational year level, stress from lack of professional knowledge and skills, stress from assignments and workload, and stress from taking care of patients, as indicated by their positive coefficients and significant p-values (p < 0.05). However, it was negatively influenced by gender (being male). The other factors do not significantly influence the surface learning approach. The adjusted R-squared values indicated that the variables in the model explain 17.8% of the variance in the deep learning approach and 25.5% in the surface learning approach. Both models were statistically significant (p < 0.001).

**Table 4:** Multivariate linear regression analysis for the parameters affecting learning approach (N = 1010)

Socio-demographic data	^#^Multivariate			
	Deep Learning approach		Surface Learning approach	
	p	B (LL– UL 95%C.I)	p	B (LL– UL 95%C.I)
Age	0.040	0.116(0.005–0.227)	0.342	0.051(-0.054-0.156)
Gender (Female /Male)	0.028	1.260(0.134–2.385)	< 0.001*	-4.039(-5.125- -2.953)
Educational year level	0.008	0.665(0.177–1.153)	< 0.001*	1.401(0.940–1.863)
Hours spent for studying	-	-	0.901	-0.030 (-0.507–0.446)
Stress from lack of professional knowledge and skills	< 0.001*	-1.178(-1.348 - -1.008)	< 0.001*	0.640 (0.479-0.800)
Stress from assignments and workload	0.979	-0.002(-0.131–0.128)	0.001*	0.217 (0.094–0.339)
Stress from taking care of patients	0.270	0.047(-0.037-0.131)	< 0.001*	0.160(0.081–0.240)
Stress from clinical environment	0.868	-0.018(-0.229-0.193)	0.720	0.036(-0.163–0.236)
Stress from teachers and nursing staff	0.020*	0.143(0.023–0.263)	0.778	0.016(-0.097- 0.130)
Stress from peers and daily life	0.748	-0.028(-0.197-0.141)	0.058	-0.154 (-0.314- 0.005)
	R^2^ = 0.185, Adj. R^2^ = 0.178, F = 25.271^*^,p < 0.001^*^		R^2^ = 0.262, Adj. R^2^ = 0.255, F = 35,457^*^,p < 0.001^*^	

B: Unstandardized Coefficients C.I: Confidence Interval LL: Lower Limit UL: Upper Limit

#: All variables with p < 0.05 was included in the multivariate *: Statistically significant at p ≤ 0.05

## Discussion

Nursing students’ academic stress and learning approaches are essential to planning for effective and efficient learning. Nursing education also aims to develop knowledgeable and competent students with problem-solving and critical-thinking skills.

The study’s findings highlight the significant presence of stress among nursing students, with a majority experiencing moderate to severe levels of academic stress. This aligns with previous research indicating that academic stress is prevalent among nursing students. For instance, Zheng et al. (2022) observed moderated stress levels in nursing students during clinical placements [23], while El-Ashry et al. (2022) found that nearly all first-year nursing students in Egypt experienced severe academic stress [21]. Conversely, Ali and El-Sherbini (2018) reported that over three-quarters of nursing students faced high academic stress. The complexity of the nursing program likely contributes to these stress levels [24].

The current study revealed that nursing students identified the highest sources of academic stress as workload from assignments and the stress of caring for patients. This aligns with Banu et al.‘s (2015) findings, where academic demands, assignments, examinations, high workload, and combining clinical work with patient interaction were cited as everyday stressors [25]. Additionally, Anaman-Torgbor et al. (2021) identified lectures, assignments, and examinations as predictors of academic stress through logistic regression analysis. These stressors may stem from nursing programs emphasizing the development of highly qualified graduates who acquire knowledge, values, and skills through classroom and clinical experiences [26].

The results regarding learning approaches indicate that most nursing students predominantly employed the deep learning approach. Despite acknowledging a surface learning approach among the participants in the present study, the prevalence of deep learning was higher. This inclination toward the deep learning approach is anticipated in nursing students due to their engagement with advanced courses, requiring retention, integration, and transfer of information at elevated levels. The deep learning approach correlates with a gratifying learning experience and contributes to higher academic achievements [3]. Moreover, the nursing program’s emphasis on active learning strategies fosters critical thinking, problem-solving, and decision-making skills. These findings align with Mahmoud et al.‘s (2019) study, reporting a significant presence (83.31%) of the deep learning approach among undergraduate nursing students at King Khalid University’s Faculty of Nursing [27]. Additionally, Mohamed &Morsi (2019) found that most nursing students at Benha University’s Faculty of Nursing embraced the deep learning approach (65.4%) compared to the surface learning approach [28].

The study observed a negative correlation between the deep learning approach and the overall mean stress score, contrasting with a positive correlation between surface learning approaches and overall stress levels. Elevated academic stress levels may diminish motivation and engagement in the learning process, potentially leading students to feel overwhelmed, disinterested, or burned out, prompting a shift toward a surface learning approach. This finding resonates with previous research indicating that nursing students who actively seek positive academic support strategies during academic stress have better prospects for success than those who do not [29]. Nebhinani et al. (2020) identified interface concerns and academic workload as significant stress-related factors. Notably, only an interest in nursing demonstrated a significant association with stress levels, with participants interested in nursing primarily employing adaptive coping strategies compared to non-interested students.

The current research reveals a statistically significant inverse relationship between different dimensions of academic stress and adopting the deep learning approach. The most substantial negative correlation was observed with stress arising from a lack of professional knowledge and skills, succeeded by stress associated with the clinical environment, assignments, and workload. Nursing students encounter diverse stressors, including delivering patient care, handling assignments and workloads, navigating challenging interactions with staff and faculty, perceived inadequacies in clinical proficiency, and facing examinations [30].

In the current study, the multivariate linear regression analysis reveals that various factors positively influence the deep learning approach, including age, female gender, educational year level, and stress from teachers and nursing staff. In contrast, stress from a lack of professional knowledge and skills exert a negative influence. Conversely, the surface learning approach is positively influenced by female gender, educational year level, stress from lack of professional knowledge and skills, stress from assignments and workload, and stress from taking care of patients, but negatively affected by male gender. The models explain 17.8% and 25.5% of the variance in the deep and surface learning approaches, respectively, and both are statistically significant. These findings underscore the intricate interplay of demographic and stress-related factors in shaping nursing students’ learning approaches. High workloads and patient care responsibilities may compel students to prioritize completing tasks over deep comprehension. This pressure could lead to a surface learning approach as students focus on meeting immediate demands rather than engaging deeply with course material. This observation aligns with the findings of Alsayed et al. (2021), who identified age, gender, and study year as significant factors influencing students’ learning approaches.

Deep learners often demonstrate better self-regulation skills, such as effective time management, goal setting, and seeking support when needed. These skills can help manage academic stress and maintain a balanced learning approach. These are supported by studies that studied the effect of coping strategies on stress levels [6, 31, 32]. On the contrary, Pacheco-Castillo et al. study (2021) found a strong significant relationship between academic stressors and students’ level of performance. That study also proved that the more academic stress a student faces, the lower their academic achievement.

### Strengths and limitations of the study

This study has lots of advantages. It provides insightful information about the educational experiences of Egyptian nursing students, a demographic that has yet to receive much research. The study’s limited generalizability to other people or nations stems from its concentration on this particular group. This might be addressed in future studies by using a more varied sample. Another drawback is the dependence on self-reported metrics, which may contain biases and mistakes. Although the cross-sectional design offers a moment-in-time view of the problem, it cannot determine causation or evaluate changes over time. To address this, longitudinal research may be carried out.

Notwithstanding these drawbacks, the study substantially contributes to the expanding knowledge of academic stress and nursing students’ learning styles. Additional research is needed to determine teaching strategies that improve deep-learning approaches among nursing students. A qualitative study is required to analyze learning approaches and factors that may influence nursing students’ selection of learning approaches.

## Conclusion

According to the present study’s findings, nursing students encounter considerable academic stress, primarily stemming from heavy assignments and workload, as well as interactions with teachers and nursing staff. Additionally, it was observed that students who experience lower levels of academic stress typically adopt a deep learning approach, whereas those facing higher stress levels tend to resort to a surface learning approach. Demographic factors such as age, gender, and educational level influence nursing students’ choice of learning approach. Specifically, female students are more inclined towards deep learning, whereas male students prefer surface learning. Moreover, deep and surface learning approach scores show an upward trend with increasing educational levels and study hours. Academic stress emerges as a significant determinant shaping the adoption of learning approaches among nursing students.

### Implications in nursing practice

Nursing programs should consider integrating stress management techniques into their curriculum. Providing students with resources and skills to cope with academic stress can improve their well-being and academic performance. Educators can incorporate teaching strategies that promote deep learning approaches, such as problem-based learning, critical thinking exercises, and active learning methods. These approaches help students engage more deeply with course material and reduce reliance on surface learning techniques. Recognizing the gender differences in learning approaches, nursing programs can offer gender-specific support services and resources. For example, providing targeted workshops or counseling services that address male and female nursing students’ unique stressors and learning needs. Implementing mentorship programs and peer support groups can create a supportive environment where students can share experiences, seek advice, and receive encouragement from their peers and faculty members. Encouraging students to reflect on their learning processes and identify effective study strategies can help them develop metacognitive skills and become more self-directed learners. Faculty members can facilitate this process by incorporating reflective exercises into the curriculum. Nursing faculty and staff should receive training on recognizing signs of academic stress among students and providing appropriate support and resources. Additionally, professional development opportunities can help educators stay updated on evidence-based teaching strategies and practical interventions for addressing student stress.

## Data Availability

The datasets generated and/or analysed during the current study are not publicly available due to restrictions imposed by the institutional review board to protect participant confidentiality, but are available from the corresponding author on reasonable request.

## References

[1] Liu J, Yang Y, Chen J, Zhang Y, Zeng Y, Li J. Stress and coping styles among nursing students during the initial period of the clinical practicum: A cross-section study. Int J Nurs Sci. 2022a;9(2). 10.1016/j.ijnss.2022.02.004. 10.1016/j.ijnss.2022.02.004 PMC9052268 35509703

[2] Saifan A, Devadas B, Daradkeh F, Abdel-Fattah H, Aljabery M, Michael LM. Solutions to bridge the theory-practice gap in nursing education in the UAE: a qualitative study. BMC Med Educ. 2021;21(1). 10.1186/s12909-021-02919-x. 10.1186/s12909-021-02919-x PMC8439067 34517861

[3] Alsayed S, Alshammari F, Pasay-an E, Dator WL. Investigating the learning approaches of students in nursing education. J Taibah Univ Med Sci. 2021;16(1). 10.1016/j.jtumed.2020.10.008. 10.1016/j.jtumed.2020.10.008 PMC7858010 33603631

[4] Salah Dogham R, Elcokany NM, Saber Ghaly A, Dawood TMA, Aldakheel FM, Llaguno MBB, Mohsen DM. Self-directed learning readiness and online learning self-efficacy among undergraduate nursing students. Int J Afr Nurs Sci. 2022;17. 10.1016/j.ijans.2022.100490.

[5] Zhao Y, Kuan HK, Chung JOK, Chan CKY, Li WHC. Students’ approaches to learning in a clinical practicum: a psychometric evaluation based on item response theory. Nurse Educ Today. 2018;66. 10.1016/j.nedt.2018.04.015. 10.1016/j.nedt.2018.04.015 29709307

[6] Huang HM, Fang YW. Stress and coping strategies of online nursing practicum courses for Taiwanese nursing students during the COVID-19 pandemic: a qualitative study. Healthcare. 2023;11(14). 10.3390/healthcare11142053. 10.3390/healthcare11142053 PMC10378767 37510494

[7] Nebhinani M, Kumar A, Parihar A, Rani R. Stress and coping strategies among undergraduate nursing students: a descriptive assessment from Western Rajasthan. Indian J Community Med. 2020;45(2). 10.4103/ijcm.IJCM_231_19. 10.4103/ijcm.IJCM_231_19 PMC7467204 32905220

[8] Olvera Alvarez HA, Provencio-Vasquez E, Slavich GM, Laurent JGC, Browning M, McKee-Lopez G, Robbins L, Spengler JD. Stress and health in nursing students: the Nurse Engagement and Wellness Study. Nurs Res. 2019;68(6). 10.1097/NNR.0000000000000383. 10.1097/NNR.0000000000000383 PMC7004871 31693551

[9] Del Giudice M, Buck CL, Chaby LE, Gormally BM, Taff CC, Thawley CJ, Vitousek MN, Wada H (2018). What is stress? A systems perspective. Integr Comp Biol.

[10] Bhui K, Dinos S, Galant-Miecznikowska M, de Jongh B, Stansfeld S. Perceptions of work stress causes and effective interventions in employees working in public, private and non-governmental organisations: a qualitative study. BJPsych Bull. 2016;40(6). 10.1192/pb.bp.115.050823. 10.1192/pb.bp.115.050823 PMC5353523 28377811

[11] Lavoie-Tremblay M, Sanzone L, Aubé T, Paquet M (2021). Sources of stress and coping strategies among undergraduate nursing students across all years. Can J Nurs Res.

[12] Ahmed WAM, Abdulla YHA, Alkhadher MA, Alshameri FA. Perceived stress and coping strategies among nursing students during the COVID-19 pandemic: a systematic review. Saudi J Health Syst Res. 2022;2(3). 10.1159/000526061.

[13] Pacheco-Castillo J, Casuso-Holgado MJ, Labajos-Manzanares MT, Moreno-Morales N. Academic stress among nursing students in a Private University at Puerto Rico, and its Association with their academic performance. Open J Nurs. 2021;11(09). 10.4236/ojn.2021.119063.

[14] Tran TTT, Nguyen NB, Luong MA, Bui THA, Phan TD, Tran VO, Ngo TH, Minas H, Nguyen TQ. Stress, anxiety and depression in clinical nurses in Vietnam: a cross-sectional survey and cluster analysis. Int J Ment Health Syst. 2019;13(1). 10.1186/s13033-018-0257-4. 10.1186/s13033-018-0257-4 PMC6317201 30622629

[15] Magnavita N, Chiorri C. Academic stress and active learning of nursing students: a cross-sectional study. Nurse Educ Today. 2018;68. 10.1016/j.nedt.2018.06.003. 10.1016/j.nedt.2018.06.003 29906771

[16] Folkvord SE, Risa CF. Factors that enhance midwifery students’ learning and development of self-efficacy in clinical placement: a systematic qualitative review. Nurse Educ Pract. 2023;66. 10.1016/j.nepr.2022.103510. 10.1016/j.nepr.2022.103510 36462273

[17] Myers SB, Sweeney AC, Popick V, Wesley K, Bordfeld A, Fingerhut R. Self-care practices and perceived stress levels among psychology graduate students. Train Educ Prof Psychol. 2012;6(1). 10.1037/a0026534.

[18] Zeb H, Arif I, Younas A. Nurse educators’ experiences of fostering undergraduate students’ ability to manage stress and demanding situations: a phenomenological inquiry. Nurse Educ Pract. 2022;65. 10.1016/j.nepr.2022.103501. 10.1016/j.nepr.2022.103501 36375443

[19] Centers for Disease Control and Prevention. User Guide| Support| Epi Info™ [Internet]. Atlanta: CDC; [cited 2024 Jan 31]. Available from: CDC website.

[20] Sheu S, Lin HS, Hwang SL, Yu PJ, Hu WY, Lou MF. The development and testing of a perceived stress scale for nursing students in clinical practice. J Nurs Res. 1997;5:41–52. Available from: http://ntur.lib.ntu.edu.tw/handle/246246/165917.

[21] El-Ashry AM, Harby SS, Ali AAG (2022). Clinical stressors as perceived by first-year nursing students of their experience at Alexandria main university hospital during the COVID-19 pandemic. Arch Psychiatr Nurs.

[22] Biggs J, Kember D, Leung DYP (2001). The revised two-factor study process questionnaire: R-SPQ-2F. Br J Educ Psychol.

[23] Zheng YX, Jiao JR, Hao WN. Stress levels of nursing students: a systematic review and meta-analysis. Med (United States). 2022;101(36). 10.1097/MD.0000000000030547. 10.1097/MD.0000000000030547 PMC10980379 36086725

[24] Ali AM, El-Sherbini HH. Academic stress and its contributing factors among faculty nursing students in Alexandria. Alexandria Scientific Nursing Journal. 2018; 20(1):163–181. Available from: https://asalexu.journals.ekb.eg/article_207756_b62caf4d7e1e7a3b292bbb3c6632a0ab.pdf.

[25] Banu P, Deb S, Vardhan V, Rao T. Perceived academic stress of university students across gender, academic streams, semesters, and academic performance. Indian J Health Wellbeing. 2015;6(3):231–235. Available from: http://www.iahrw.com/index.php/home/journal_detail/19#list.

[26] Anaman-Torgbor JA, Tarkang E, Adedia D, Attah OM, Evans A, Sabina N (2021). Academic-related stress among Ghanaian nursing students. Florence Nightingale J Nurs.

[27] Mahmoud HG, Ahmed KE, Ibrahim EA. Learning Styles and Learning Approaches of Bachelor Nursing Students and its Relation to Their Achievement. Int J Nurs Didact. 2019;9(03):11–20. Available from: http://www.nursingdidactics.com/index.php/ijnd/article/view/2465.

[28] Mohamed NAAA, Morsi MES, Learning Styles L, Approaches (2019). Academic achievement factors, and self efficacy among nursing students. Int J Novel Res Healthc Nurs.

[29] Onieva-Zafra MD, Fernández-Muñoz JJ, Fernández-Martínez E, García-Sánchez FJ, Abreu-Sánchez A, Parra-Fernández ML (2020). Anxiety, perceived stress and coping strategies in nursing students: a cross-sectional, correlational, descriptive study. BMC Med Educ.

[30] Aljohani W, Banakhar M, Sharif L, Alsaggaf F, Felemban O, Wright R. Sources of stress among Saudi Arabian nursing students: a cross-sectional study. Int J Environ Res Public Health. 2021;18(22). 10.3390/ijerph182211958. 10.3390/ijerph182211958 PMC8624098 34831714

[31] Liu Y, Wang L, Shao H, Han P, Jiang J, Duan X. Nursing students’ experience during their practicum in an intensive care unit: a qualitative meta-synthesis. Front Public Health. 2022;10. 10.3389/fpubh.2022.974244. 10.3389/fpubh.2022.974244 PMC9556851 36249222

[32] Majrashi A, Khalil A, Nagshabandi E, Al MA (2021). Stressors and coping strategies among nursing students during the COVID-19 pandemic: scoping review. Nurs Rep.

